# Tratamento da dor lombar com uma combinação de trifosfato de uridina, monofosfato de citidina e hidroxicobalamina: Uma revisão sistemática e metanálise

**DOI:** 10.1055/s-0045-1809521

**Published:** 2025-07-10

**Authors:** Marco Antonio N. Mibielli, Mendel Suchmacher, Mauro Geller, Spyros G. E. Mezitis, Carlos P. Nunes, Aline Sintoveter

**Affiliations:** 1 Centro Universitário Serra dos Órgãos, Teresópolis, RJ, Brasil; 2 Programa de Pós-graduação em Imunologia, Instituto de Pós-graduação Médica Carlos Chagas, Rio de Janeiro, RJ, Brasil; 3 Endocrinologia e Medicina Interna, Cornell University Medical School, Nova York, NY, Estados Unidos; 4 University of Central Florida College of Medicine, Orlando, FL, Estados Unidos

**Keywords:** dor lombar, hidroxicobalamina, monofosfato de citidina, raízes nervosas espinhais, uridina trifosfato, cytidine monophosphate, hydroxocobalamin, low back pain, spinal nerve roots, uridine triphosphate

## Abstract

A dor lombar é uma queixa comum. Essa síndrome tem diferentes mecanismos subjacentes, difíceis de diferenciar em tempo hábil apenas por meio dos recursos semióticos, laboratoriais e de imagem disponíveis no atendimento de emergência. Isso faz com que os profissionais tendam a uma abordagem sintomática inicial composta por medicamentos (anti-inflamatórios não esteroidais, analgésicos, relaxantes musculares) ou procedimentos locais (calor local, massagem). Substâncias neurotróficas periféricas, como nucleotídeos de pirimidina (trifosfato de uridina e monofosfato de citidina) combinados com vitamina B12 (hidroxicobalamina), têm sido usadas como precursores anabólicos capazes de fornecer às raízes nervosas espinhais elementos desencadeadores úteis para a regeneração de neurônios e células da glia, uma vez que um provável mecanismo de compressão espinhal seja contido. Os autores realizaram uma revisão sistemática e meta-análise com a combinação acima com o objetivo de determinar melhor seu papel no tratamento da dor lombar.

## Abstract (en)

Low back pain is a common complaint. This syndrome comprehends different underlying mechanisms, which are difficult to differentiate in a timely manner only through semiotic, laboratory, and imaging resources available in an emergency setting. Such circumstances make practitioners prone to an initial symptomatic approach in the form of medications (non-steroid anti-inflammatory drugs, analgesics, muscle relaxants) or local procedures (local heat, massage). Peripheral neurotrophic substances, such as pyrimidine nucleotides (uridine triphosphate and cytidine monophosphate) combined with vitamin B12 (hydroxocobalamin), have been used as anabolic precursors able to provide spinal nerve roots with triggering elements useful for nerve and glial cells regeneration, once a likely spinal compression mechanism is contained. The authors performed a systematic review and meta-analysis with the above combination with the aim of better determining its role in low back pain management.

## Introdução

A dor lombar (dor lombossacral) associada às síndromes de neuropatia compressiva representa uma das manifestações patológicas mais frequentes da coluna vertebral. A dor lombar é um termo pouco específico que pode compreender distintas entidades clinicamente expressas de forma isolada, combinada ou sobreposta. 1 Sua prevalência varia de 30 a 70% na população de 18 a 74 anos. 2 A ciática, por sua vez, é um termo geral usado para se referir à dor radicular lombar, geralmente com irradiação unilateral para a perna de acordo com o dermátomo correspondente. Pode ser acompanhada por déficits motores, sensitivos e/ou de reflexos. A dor é pior do que a dor lombar “clássica” e o risco de cronicidade é maior. 3 Várias modalidades terapêuticas—conservadoras, farmacológicas e invasivas—foram desenvolvidas e aplicadas ao longo dos últimos 100 anos. A combinação de trifosfato de uridina (UTP), monofosfato de citidina (CMP) e hidroxicobalamina foi prescrita em alguns países para o controle sintomático desta síndrome nos últimos 50 anos. O objetivo desta revisão sistemática e metanálise é medir os efeitos da combinação neste cenário.

## Materiais e Métodos

### Pesquisa e Seleção dos Estudos Primários

**Fig. 1:**
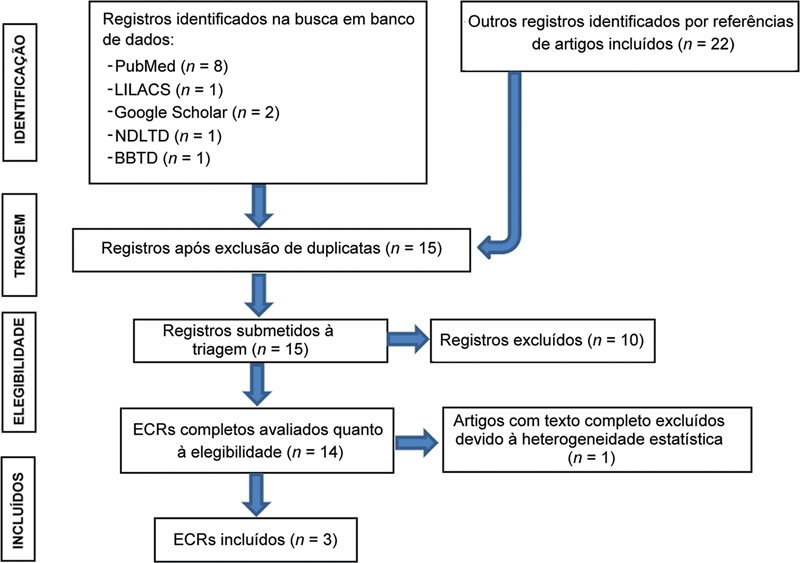
Diagrama para seleção de estudos aplicado nesta revisão sistemática e meta-análise. **Abreviaturas:** LILACS, Literatura Latino-Americana e do Caribe em Ciências da Saúde; NDLTD, Networked Digital Library of Theses and Dissertations; BBTD, Biblioteca Digital Brasileira de Teses e Dissertações; ECR, ensaio clínico controlado.

O estudo foi realizado por dois pesquisadores independentes que trabalharam em paralelo e às cegas, ambos de acordo com os seguintes parâmetros: (1) estudos epidemiológicos, estudos observacionais, ensaios clínicos randomizados (ECRs) ou não (não ECR), revisões sistemáticas e metanálises; (2) nenhuma restrição de idioma ou ano de publicação; e (3) sem considerar os nomes dos autores dos estudos primários (embora a consulta pessoal fosse permitida). A literatura de apoio, como livros didáticos, artigos científicos básicos e compêndios farmacológicos, foi consultada quando necessário, mas não contabilizada para fins de revisão sistemática. A busca de estudos foi realizada de acordo com as diretrizes da declaração Preferred Reporting Items for Systematic Reviews and Meta-analyses (PRISMA). 4 A Fig. 1 mostra o fluxograma e o Apêndice detalha as fontes analisadas.

**Apêndice 1:** Fontes analisadas e seus parâmetros

1. Pubmed: (1) *uridine triphosphate* , UTP, *cytidine monophosphate* , CMP, *hydroxocobalamin* , *vitamin B12* , *spinal nerve root* , e *pyrimidine nucleotides* no título, e/ou (2) *low back pain* , lumbago, lumbopelvic pain, ou sciatica em qualquer parte do texto; 2. LILACS: (1) uridina trifosfato, UTP, citidina monofosfato, CMP, hidroxocobalamina, vitamina B12, raiz dorsal, nucleotídeos pirimidínicos no título, e (2) lombalgia, dor lombar ou ciatalgia em qualquer parte do texto; 3. Google Scholar: (1) *uridine triphosphate* , UTP, *cytidine monophosphate* , CMP, *hydroxocobalamin* , *vitamin B12* , *spinal nerve root* , e *pyrimidine nucleotides* no título, e/ou (2) *low back pain* , *lumbago* , *lumbopelvic pain* , ou *sciatica* em qualquer parte do texto, e (3) até três páginas de pesquisa; 4. NDLTD: *uridine triphosphate* , UTP, *cytidine monophosphate* , CMP, *hydroxocobalamin* , *vitamin B12* , *spinal nerve root* , e *pyrimidine nucleotides* no título; 5. BBTD: uridina trifosfato, UTP, citidina monofosfato, CMP, hidroxicobalamina, vitamina B12, raiz dorsal ou nucleotídeos pirimidínicos no título; 6. Referências bibliográficas de publicações selecionadas.

NDLTD, *Networked Digital Library of Theses and Dissertations* ; BBTD, Biblioteca Digital Brasileira de Teses e Dissertações.

### Coleta de Resultados e Desfechos

Os resultados dos dois pesquisadores foram cruzados por um revisor para validação, que não relatou conflitos entre o corpo de achados dos dois primeiros profissionais. Os estudos foram selecionados de acordo com seus respectivos títulos e resumos, de acordo com os seguintes parâmetros de interesse: (1) dor lombar, (2) combinação de UTP, CMP e hidroxicobalamina em seu controle sintomático, (3) comparação de eficácia e segurança com vitamina B12 (hidroxicobalamina e cianocobalamina) e (4) propriedades farmacológicas aditivas e/ou sinérgicas da combinação de UTP, CMP e hidroxicobalamina. A busca textual foi estendida do título/resumo para o corpo do texto caso necessário. Não houve necessidade de contato pessoal com os autores dos estudos. Uma literatura abrangente sobre a farmacologia geral de UTP, CMP e hidroxicobalamina também foi consultada.

Este estudo seguiu as diretrizes PRISMA. 4 As medidas da Escala Visual Analógica (EVA) foram escolhidas como desfecho do estudo. A diferença média entre a EVA basal e final, juntamente com o desvio padrão, foram usados para o grupo UTP, CMP e vitamina B12, bem como o grupo de monoterapia com vitamina B12, com intervalos de confiança (ICs) de 95%. O teste Q de Cochran e as estatísticas I2 foram usados para avaliação da heterogeneidade. Valores de *p* < 0,10 e I2 ≥ 50% foram considerados para determinar a significância da heterogeneidade e o uso de um modelo de efeito aleatório. Um dos autores conduziu as análises estatísticas usando o programa R (R Foundation for Statistical Computing, Viena, Áustria). 5

## Lesões das Raízes Nervosas Espinhais e Dor Lombar Crônica

### Definição

A dor lombar pode ser definida como uma dor na linha média que se estende da costela mais inferior até a prega glútea. Essa síndrome pode ser classificada como aguda (< 6 semanas), subaguda (6–12 semanas) ou crônica (> 12 semanas de duração). Pode ser acompanhada por perda de mobilidade, irradiação para as pernas, virilha e pelve posterior, alteração de humor e distúrbios nas interações sociais. 1 2

### Fisiopatologia da Dor Lombar

A dor lombar é o resultado de um processo fisiopatológico complexo com acometimento das estruturas nervosas (raízes nervosas espinhais e gânglios da raiz dorsal) e somáticas (articulações facetárias intervertebrais, periósteo, ligamentos, tendões, fáscias, músculos paravertebrais e discos intervertebrais). 6 Na maioria dos casos, os dois mecanismos se sobrepõem. Embora uma estrutura culpada não possa ser apontada em 80 a 90% dos casos, o acometimento do tecido nervoso pode ser observado em 10 a 15% dos pacientes. 1 2 Hérnias de disco, doenças degenerativas crônicas da coluna e estenose espinhal são os mecanismos etiológicos mais comuns encontrados na prática clínica. 1 6 A compressão mecânica aguda exercida por um pedículo vertebral sobre uma raiz nervosa espinhal lombossacral e/ou gânglio da raiz dorsal pode causar comprometimento vascular local, alterações microestruturais e inflamação, todos ligados a déficits sensoriais, diminuição do limiar de dor e perda de força somática e controle autonômico (bexiga e intestino) no dermátomo correspondente. 7 O início da dor é proporcional ao grau de compressão extrínseca e irritação da raiz nervosa espinhal, ambos difíceis de determinar no ambiente clínico. 6 8 Durante a recuperação, citocinas inflamatórias locais e fatores neurotróficos liberados para a cicatrização do tecido neural podem diminuir o limiar da dor, promovendo a neuroplasticidade e estimulando neurônios vizinhos não lesionados, com piora álgica paradoxal. 8

### Quadro Clínico, Diagnóstico e Prognóstico

Caso presente, a dor neuropática se manifesta como parestesia, hiperestesia, alodinia e hiperalgesia. A identificação da origem estrutural da dor pode ser difícil, já que os mecanismos fisiopatológicos se sobrepõem e evoluem de maneira dinâmica. Da mesma forma, manobras como compressão digital e testes de mobilidade são limitadas devido à inacessibilidade semiótica das estruturas possivelmente acometidas e ao baixo poder discriminatório. 1 2 Os diagnósticos diferenciais de dor lombar são fratura local (trauma, queda de altura, osteoporose preexistente), infecções (sintomas B [febre, sudorese, dor noturna], imunossupressão, abuso de drogas intravenosas) ou doença tumoral (sintomas B, dor exacerbada na posição supina, paraproteinemia). 2 Os exames de diagnóstico por imagem são, de modo geral, desnecessários nos primeiros estágios da apresentação da dor lombar. No entanto, se indicados, o médico assistente deve levar em consideração que a disfunção segmentar e muscular, bem como a síndrome da articulação sacroilíaca, não são passíveis de demonstração morfológica. 2 6 A dor lombar resolve-se em 4 a 6 semanas em 50% dos casos e em 12 semanas em 80% dos casos. Por outro lado, a recidiva e a incapacidade de trabalhar são características comuns se a etiologia não for abordada. 2

### Neurorregeneração e Dor Lombossacral

Os gânglios da raiz dorsal têm axônios que alcançam lateral e medialmente as raízes nervosas espinhais e a medula espinhal, respectivamente. Lesões nas raízes nervosas espinhais induzem um processo regenerativo, semelhante a lesões neuropáticas compressivas, enquanto lesões na medula espinhal não se regeneram devido ao ambiente inibidor do sistema nervoso central (SNC). 6 Presumindo que os axônios das raízes nervosas espinhais apresentem uma biologia semelhante à dos nervos periféricos, é possível supor que ambos podem compartilhar padrões regenerativos semelhantes. Portanto, uma classificação combinada de Seddon e Sunderland de lesões nervosas periféricas poderia ser proposta para classificar lesões de raízes nervosas espinhais no contexto de dor lombar ( Tabela 1 ).

**Tabela 1:** Classificação combinada de Seddon e Sunderland de lesões de nervos periféricos 9

Sunderland	Seddon	Fisiopatologia	Recuperação
Primeiro grau	Neuropraxia	Desmielinização segmentar	Completa
Segundo grau	Axonotmese	Axônio rompido, endoneuro intacto	Completa
Terceiro grau	−	Axônio rompido, endoneuro comprometido perineuro intacto	Variável
Quarto grau	−	Perda de continuidade de axônio, endoneuro e perineuro, epineuro intacto	Nula
Quinto grau	Neurotmese	Perda de continuidade de todo o nervo	Nula

### Tratamento e Prognóstico

As medidas conservadoras incluem fisioterapia (tração espinhal, alongamento, massagem), técnicas de relaxamento, terapia cognitivo-comportamental, estimulação elétrica nervosa transcutânea ( *transcutaneous electrical nerve stimulation* , TENS, em inglês), exercícios, repouso em leito ou simplesmente a retomada das atividades diárias normais. A prevenção de contraturas musculares é fundamental, pois podem atrasar a recuperação funcional. O tratamento médico do componente nevrálgico da síndrome inclui antidepressivos (gabapentina, oxcarbazepina e lamotrigina) e pregabalina. O componente de dor somática pode ser tratado com paracetamol ou anti-inflamatórios não esteroidais (ibuprofeno, diclofenaco, naproxeno). 1 2 6 A cicatrização de uma lesão mecânica contra uma raiz nervosa espinhal depende da estrutura específica envolvida, do grau de insulto, do seu mecanismo e duração. Mesmo com o tratamento ideal, a recuperação é tipicamente incompleta e pode haver persistência da dor disfuncional e neuropática. Hoje, não há fatores prognósticos reconhecíveis para recuperação motora ou resolução da dor associada a esses tipos de lesão. 8 9 10

## Nucleotídeos de Pirimidina – UTP e CMP

**Fig. 2:**
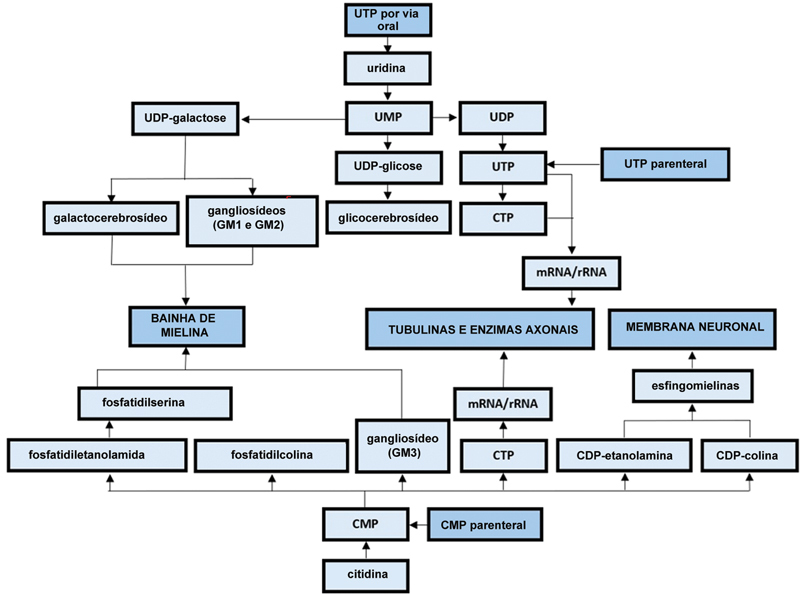
Vias bioquímicas de células nervosas e células de Schwann passíveis de influência farmacológica por nucleotídeos de pirimidina.

O modelo estrutural de nucleotídeos tem sido usado no desenvolvimento de várias substâncias farmacologicamente ativas, como metabólitos antitumorais (por exemplo, mercaptopurina), análogos de nucleosídeos (por exemplo, lamivudina) e antiarrítmicos de nucleosídeos (por exemplo, adenosina). No contexto de neuropatias de compressão, os nucleotídeos de pirimidina UTP e CMP são usados como substâncias neurotróficas metabólicas envolvidas na estimulação da síntese da membrana neuronal, da bainha de mielina e de proteínas axonais (tubulinas e enzimas) ( Fig. 2 ).

### Farmacodinâmica dos Nucleotídeos de Pirimidina

**Aumento na síntese de proteínas das células nervosas.** Espera-se a ocorrência de degeneração Walleriana após a desintegração dos axônios e células de Schwann por um trauma mecânico em um nervo periférico ou célula da raiz nervosa. A velocidade das vias anabólicas soma e mielínica é correspondentemente acelerada durante esse fenômeno. Portanto, espera-se que a quantidade de consumo de nucleotídeos seja maior em comparação às células nervosas e da glia em seu estado estável, juntamente com outros metabólitos vitais. 11 12 13 14

**Aumento na síntese da bainha de mielina.** Nos estágios posteriores da degeneração Walleriana, a passagem de cones de regeneração axonal através da banda de Bungner no coto neural distal desencadeia o envolvimento da membrana das células de Schwann ao redor dos axônios em avanço, formando novas bainhas de mielina. Espera-se que a demanda por precursores de lipídios de mielina nas células de Schwann, inclusive metabólitos intermediários de nucleotídeos, seja aumentada sob as condições acima. 13 15 16

**Aumento da síntese da membrana da célula nervosa.** Similar ao mecanismo de aumento da síntese da bainha de mielina, um aumento nos elementos estruturais axolemais deve ocorrer em paralelo à progressão dos cones regenerativos. A integração de intermediários metabólicos de nucleotídeos *de novo* neste cenário também é necessária. 14 16 17 18 19 20 21 22 23

### Segurança dos Nucleotídeos de Pirimidina

A UTP e a CMP são contraindicadas na fase aguda do acidente vascular cerebral isquêmico devido à possibilidade de degradação da fosfatidilcolina da membrana das células nervosas em diacilglicerol e ácidos graxos livres em condições de anóxia cerebral. 24 25 Indivíduos com deficiências de diidropirimidina desidrogenase ou ornitina carbamoil transferase podem apresentar excesso de nucleotídeos de pirimidina no SNC. Portanto, UTP e CMP são contraindicadas em pacientes com as condições acima descritas. 16

## Hidroxicobalamina

A hidroxicobalamina é uma forma sintetizada injetável de vitamina B12. Participa nas chamadas reações do metabolismo de um carbono (síntese de nucleotídeos de cisteína, metionina e pirimidina, bem como reações de metilação), metabolismo mitocondrial e síntese de proteína básica de mielina (MBP; estabilização estrutural da bainha de mielina). As propriedades analgésicas da vitamina B12 ainda são debatidas, mas aparentemente, a vitamina acelera a melhora da dor lombar devido à sua participação na recuperação da bainha de mielina. 26 As reações adversas da hidroxicobalamina são eritema acneiforme, febre, ondas de calor exantemáticas, hipertensão arterial (injeções intravenosas), edema periférico, fotossensibilidade, prurido e urticária (relatos de casos). 27 28 29

As síndromes de compressão da raiz nervosa espinhal apresentam fisiopatologia complexa, sugerindo possíveis alvos para diferentes modalidades terapêuticas. Como mostra a Fig. 2 , a UTP e a CMP compartilham efeitos sinérgicos sobre as vias metabólicas regeneradoras do nervo periférico, cujo braço de síntese da bainha de mielina pode ser influenciado de forma aditiva pela hidroxicobalamina ao promover a síntese de MBP. Assumindo que a dor lombar neuropática também pode ser desencadeada pela desintegração das microestruturas do nervo e das células de Schwann da raiz nervosa espinhal, pode-se presumir que sua ressíntese antecipada poderia levar a uma melhora mais rápida da dor.

## Resultados

Obtivemos um total de 30 estudos gerais e 5 estudos clínicos sobre UTP, CMP e hidroxicobalamina (4 ECRs e 1 não ECR) sobre o tratamento da dor lombar, os últimos abrangendo um total de 1.236 pacientes (não encontramos nenhum estudo epidemiológico, estudo observacional, revisão sistemática ou meta-análise). Os desfechos de pesquisa relatados foram: (1) EVA (desfecho selecionado para meta-análise como uma escala visual de 0–100 mm), (2) Questionário de Funcionalidade do Paciente ( *Patient Functionality Questionnaire* - PFQ, em inglês), (3) porcentagem de pacientes com melhora no PFQ, (4) porcentagem de pacientes apresentando melhora na EVA, (5) avaliação global do paciente, (6) avaliação global do médico, (7) Questionário de Roland-Morris e (8) distância do dedo ao chão. A combinação foi considerada eficaz na redução da EVA do que um comparativo em três ECRs, 30 31 32 menos eficaz que a combinação de diclofenaco e colestiramina em um ECR 26 e eficaz em um estudo autopareado. 32 Todos os cinco estudos consideraram a combinação segura. Os achados desses estudos estão resumidos na Tabela 2 .

**Tabela 2:** Ensaios clínicos randomizados (ECRs) selecionados, não ECRs e estudo autopareado sobre a combinação de trifosfato de uridina (UTP), monofosfato de citidina (CMP) e hidroxicobalamina* no tratamento de síndromes de compressão do nervo espinhal

Autores	Objetivos do estudo	Esquemas	Tipo de estudo	*n*	Resultados	Segurança	Conclusão
**Goldberg et al.** 29 **(2009)**	Avaliar uma combinação de UTP, CMP e hidroxicobalamina no tratamento de neuralgia por alterações degenerativas da coluna ortopédica com compressão neural	Grupo A: 2 cápsulas de UTP, CMP e hidroxicobalamina. Grupo B: 2 cápsulas de hidroxicobalamina 1.000 mcg. Ambos os esquemas 3 vezes ao dia por 30 dias	Duplo-cego e randomizado	*n*_A _ = 40 *n*_B _ = 40	Houve redução da EVA em ambos os grupos, embora significativamente maior no grupo A ( *p* < 0,0001)	Os eventos adversos foram considerados leves a moderados, com desempenho geral estatisticamente melhor no grupo B	A combinação do grupo A apresentou efeito positivo no parâmetro dor nas alterações ortopédicas degenerativas da coluna com compressão neural
**Mibielli et al.** 26 **(2010)**	Avaliar a eficácia e a segurança de UTP, CMP e hidroxicobalamina no tratamento de dor aguda e não traumática na região lombar, do quadril e cervical	Grupo A: (1) pacote A contendo 6 cápsulas de UTP, CMP e hidroxicobalamina, e (2) pacote B contendo 2 cápsulas de diclofenaco-colestiramina. Grupo B: (1) pacote A contendo 6 cápsulas de UTP, CMP e hidroxicobalamina, e (2) pacote B contendo 2 cápsulas de placebo. Pacote A tomado como 2 cápsulas 3 vezes ao dia e pacote B como 1 cápsula 2 vezes ao dia por dia, ambos os grupos durante 10 dias.	Duplo-cego e randomizado	*n*_A _ = 40 *n*_B _ = 41	A combinação do grupo A resultou em um maior número de indivíduos com redução da pontuação EVA > 30 mm em comparação ao grupo B ( *p* < 0,0006)	O número de indivíduos que apresentaram eventos adversos não variou significativamente entre os grupos	A combinação do grupo A reduziu a dor entre indivíduos com dor não traumática na região lombar, dos quadris e cervical
**Mibielli et al.** 32 **(2014)**	Corroborar os efeitos analgésicos de UTP, CMP e hidroxicobalamina observados no Grupo B do estudo de Mibielli et al. 26 (2010)	Pacote A contendo 6 cápsulas de UTP, CMP e hidroxicobalamina tomadas como 2 cápsulas três vezes ao dia. Pacote B contendo 2 cápsulas de placebo tomadas como uma cápsula duas vezes ao dia durante 10 dias	Autopareado	*N* = 41	A diferença entre o 10° dia de terapia e o escore EVA pré-tratamento foi estatisticamente significativa ( *p* < 0,0001)	Idem Mibielli et al. (2010)	A combinação do grupo B parece ter propriedades analgésicas em uso de médio prazo em dores agudas e não traumáticas na região lombar, dos quadris e cervical
**Goldberg et al.** 30 **(2017)**	Avaliar a segurança e eficácia da combinação UTP, CMP e hidroxicobalamina em pacientes com neuralgia por alterações ortopédicas degenerativas e traumas (síndrome da região lombar, do quadril e do túnel do carpo) associados à compressão neural	Grupo A: 2 cápsulas de UTP, CMP e hidroxicobalamina. Grupo B: 2 cápsulas de hidroxicobalamina 1.000 mcg. Ambos os esquemas três vezes ao dia por 30 dias	Duplo-cego e randomizado	*n*_A _ = 200 *n*_B _ = 200	Houve uma superioridade estatisticamente significativa no esquema do grupo A na redução da EVA ( *p* = 0,0003)	Ambos os grupos apresentaram eventos adversos transitórios, mas nenhum evento adverso grave	A combinação do grupo A foi segura e eficaz no tratamento de neuralgias por alterações ortopédicas degenerativas associadas à compressão neural
**Mibielli et al.** 31 **(2020)**	Comparar a eficácia e tolerabilidade da combinação de UTP, CMP e hidroxicobalamina com a combinação de tiamina, piridoxina e cianocobalamina em pacientes com dor lombar	Grupo A: 2 cápsulas de UTP, CMP e hidroxicobalamina. Grupo B: 2 cápsulas de tiamina, piridoxina e cianocobalamina. Ambos os esquemas três vezes ao dia por 60 dias	Duplo-cego e randomizado	*n*_A _ = 317 *n*_B _ = 317	A redução da pontuação EVA foi estatisticamente significativa em ambos os grupos nos dias 30 e 60 ( *p* < 0,0001), com desempenho comparativamente melhor da combinação do grupo A no dia 30 ( *p* < 0,001)	75 (24%) e 105 (33%) dos indivíduos apresentaram efeitos adversos nos grupos A e B, respectivamente	A redução da pontuação EVA foi documentada em ambas as combinações dos grupos aos dias 30 e 60, com desempenho comparativamente melhor da combinação do grupo A no dia 30

**Nota:** *As cápsulas da combinação continham 1,5 mg, 2,5 mg e 1.000 mcg de UTP, CMP e hidroxicobalamina, respectivamente.

**Fig. 3:**
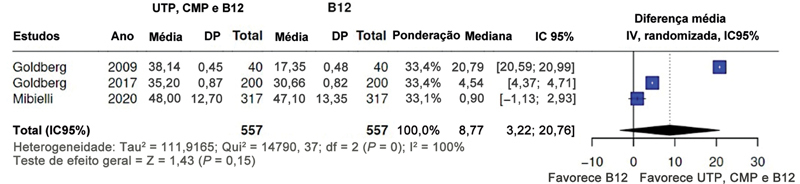
Metanálise sobre a combinação de trifosfato de uridina (UTP), monofosfato de citidina (CMP) e hidroxicobalamina (B12) no controle da dor lombar. O gráfico de floresta favorece a combinação em relação à vitamina B12. **Abreviaturas:** IC, intervalo de confiança; df, *degrees of freedom* (graus de liberdade); DP, desvio padrão; IV, ponderada.

Dos 5 estudos sobre a combinação de UTP, CMP e hidroxicobalamina detalhados na Tabela 2 , 3 apresentaram resultados comparáveis para metanálise (diferenças médias da escala EVA para a combinação *versus* hidroxicobalamina) (mediana: 8,77; IC95%: -3,22–20,76). A análise combinada dos estudos primários e a representação correspondente em gráfico de floresta são apresentadas na Fig. 3 .

## Discussão

As síndromes de dor lombar frequentemente são crônicas e comprometem o bem-estar, a produtividade pessoal e a qualidade de vida dos pacientes. Sua fisiopatologia complexa faz com que uma abordagem terapêutica multialvo viável e a associação de combinações medicamentosas é uma modalidade plausível. A combinação de UTP, CMP e hidroxicobalamina mostrou evidências de sua eficácia e segurança no controle da dor lombar com possível compressão da raiz nervosa espinhal em vários ECRs e nesta atual revisão sistemática e metanálise. Sua combinação neste cenário tem uma justificativa fisiopatológica por meio de efeitos aditivos e sinérgicos. Uma limitação do nosso estudo foi o número limitado de ECRs passíveis de metanálise. Não obstante, consideramos que nossos achados justificam a combinação de UTP, CMP e hidroxicobalamina como um recurso que pode ser útil no tratamento da dor lombar associada à compressão da raiz nervosa espinhal.

## Conclusão

Com base nos resultados desta revisão sistemática e metanálise, consideramos que a combinação de UTP, CMP e hidroxicobalamina para o tratamento da dor lombar associada à compressão da raiz nervosa espinhal é segura e eficaz.
